# Extreme Values and Convergence of the Voronoi Entropy for 2D Random Point Processes and for Long-Range Order

**DOI:** 10.3390/e28010095

**Published:** 2026-01-13

**Authors:** Mark Frenkel, Irina Legchenkova, Edward Bormashenko, Shraga Shoval, Michael Nosonovsky

**Affiliations:** 1 Department of Chemical Engineering, Ariel University, P.O.B. 3, Ariel 407000, Israel; markfr@ariel.ac.il (M.F.); lehchenko@vutbr.cz (I.L.); edward@ariel.ac.il (E.B.); 2 CEITEC BUT, Brno University of Technology, Purkyňova 123, 61200 Brno, Czech Republic; 3 Department of Industrial Engineering and Management, Faculty of Engineering, Ariel University, P.O.B. 3, Ariel 407000, Israel; shraga@ariel.ac.il; 4 Department of Mechanical Engineering, University of Wisconsin-Milwaukee, Milwaukee, WI 53211, USA

**Keywords:** Voronoi entropy, Shannon entropy, random point process, hyperuniformity

## Abstract

We investigate the asymptotic maximum value and convergence of the Voronoi Entropy (VE) for a 2D random point process (*S* = 1.690 ± 0.001) and point sets with long-range order characterized by hyperuniformity. We find that for the number of polygons of about *n* > 100, the VE range is between S = 0 (ordered set of seed points) and S = 1.69 (random set of seed points). For circular regions with the dimensionless radius R normalized by the average distance between points, we identify two limits: *Limit-1* (R = 2.5, 16 ± 6 points) is the minimum radius, for which it is possible to construct a Voronoi diagram, and *Limit-2* (R = 5.5, 96 ± 6 points) at which the VE reaches the saturation level. We also discuss examples of seed point patterns for which the values of VE exceed the asymptotic value of S > 1.69. While the VE accounts only for neighboring polygons, covering the 2D plane imposes constraints on the number of polygons and the number of edges in polygons. Consequently, unlike the conventional Shannon Entropy, the VE captures some long-range order properties of the system. We calculate the VE for several hyperuniform sets of points and compare it with the values of exponents of collective density variables characterizing long-range correlations in the system. We show that the VE correlates with the latter up to a certain saturation level, after which the value of the VE falls to *S* = 0, and we explain this phenomenon.

## 1. Introduction

The Voronoi tessellation of a set of points on a 2D plane and calculation of the Voronoi Entropy (VE) is a common method of estimating quantitatively the orderliness of various 2D systems including colloid crystals [1], self-assembled droplet clusters [2,3], surfaces of self-healing materials, molecular [4] and supramolecular [5] systems, and polymer breath figures [6]. Due to the ease of its calculation and availability in many standard software packages, the VE is widely used as a measure to estimate the orderliness of self-assembled patterns, along with other measures such as the Continuous Symmetry Measure (CSM) [7], and collective density variables [8,9].

An infinite plane with a set of seed points is partitioned into polygons based on the distance to each seed point. For each seed, there is a corresponding polygonal region consisting of all points closer to that seed than to any other. Such partition of a 2D plane into polygons is called a Voronoi tessellation or a Voronoi diagram.

The VE is the entropy of the Voronoi tessellation calculated as

(1) $$S=-\sum_{n=3}^{\infty }p_{n}\ln\left(p_{n}\right)$$

where *n* is the number of edges, $p_{n}$ is the fraction of polygons with *n* edges in the Voronoi diagram [2]. The VE is the Shannon Entropy (also known as Shannon’s Measure of Information [3,4]) of the Voronoi Tessellation. The Shannon Measure of Information is an average measure of the unlikelihood [4]. The VE, in turn, is a quantile parameter that characterizes the diversity of polygons in each tessellation, which is often seen as a measure of the orderliness of the pattern. For an ordered pattern that consists of the same type of polygons, the value of VE is *S* = 0 [5].

For a random set of points on a 2D plane (also called the random point process), it was claimed in the literature that the value of the VE is *S* = 1.71 [8]. Unfortunately, the authors of Ref. [8] do not provide a literary source or a description of the procedure for obtaining this value. Our calculations based on the data presented in work [9,10] which took into account the eleven types of polygons, from triangles to 13-gons, give a value close to *S* = 1.690 ± 0.001 (Table 1).

Finding the frequency distribution of polygons with different numbers of edges for a random point process is a challenging problem of stochastic geometry [11,12,13,14,15,16,17,18,19]. Hayen and Quine suggested an analytical solution for *n* = 3 [17], and Calca for the general case of any integer *n* ≥ 3 [18], these solutions are in the integral form, which requires numerical integration. Theoretical analysis suggests that a generalized three-parameter Gamma (3P) distribution provides the frequency distribution of the number of sides [11,13]. The distribution peaks at *n* = 6 and behaves as *p_n_* ~*N*^−2n^ for large *n* [2]. Moreover, using the Euler identity, which relates the total number of vertices, *V*, edges, *M*, and polygons, *N*, as V−M+N=1, and that there are three edges per vertex but an edge connects two vertices, 2M=3V, for large patterns, one finds the total number of edges *M* = 3*N*. In other words, the average number of edges in a polygon is *n* = 6. Note that this result is valid under certain assumptions, such as that only three edges meet at any vertices (this may be incorrect in some degenerate cases, for example, tiling by squares or by rectangles of equal sizes), a fractal set of points can also result in a deviation from the $\langle n\rangle =6$ average.

The occurrence of polygons with a larger number of edges in a random pattern will not significantly change this number, since the frequency of such polygons is negligible.

Thus, one can expect that the correction to the value *S* = 1.690 considering 14-gons will not exceed 0.00005, and for 15-gons will be only about 0.00001. For this reason, the asymptotic value of *S* = 1.690 ± 0.001 is considered the average value of the entropy of a Voronoi random set of points [15].

While the value of the VE is often in the range between S = 0 (for an ordered pattern) and *S* = 1.69 (for a random pattern), in certain situations, it can attain higher values. Moreover, the convergence rate of the VE to the asymptotic value should be the subject of investigation. It has been suggested that density oscillations of material could be related to the definition of clusters, as an intermediate level between discrete molecules and continuum matter [20] and even to the Planck mass (on the order of 10^−8^ kg) as a boundary of the macroscale (gravitational) and microscale (quantum) forces, as well as to aperiodic crystals and quasicrystals [21,22,23,24,25,26]. Defining such a boundary between the scales, based on convergence, while is a bit speculative, constitutes a challenging problem. These issues are the subject of the present study.

The Continuous Symmetry Measure (CSM) [9] for a set of m points, denoted by *ψ*(G) is based on measuring the deviation of an approximately symmetrical set from the exact symmetry, usually employing the sum of squares of the distances between the symmetric and disturbed position of the points

(2) $$\psi =\frac{1}{mR_{g}^{2}}\sum_{i=1}^{m}{\left|\vec{M_{i}}-\vec{M_{0i}}\right|}^{2}$$

where $\vec{M_{i}}$ is the position of the *i*-th point, and $\vec{M_{0i}}$ is its position in the symmetric set subject to some symmetry group *G*, and *R_g_* is the radius of gyration about the center of mass of the symmetric set. The CSM is often interpreted as a “minimal effort” required for the transformation of an original shape into a symmetric one. For a Voronoi tessellation, CSM can be defined as the average of CSM’s of all polygonal cells [27].

The CSM was successfully used for the study of approximately symmetric molecules. A comparison of the VE and the CSM was conducted by Frenkel et al. for levitating self-assembled droplet clusters [27]. They showed that the maxima and minima of the VE and CSM are not always well correlated. Symmetry and orderliness of 2D patterns could not be quantified with a single mathematical measure.

Density variables are used to characterize hyperuniform systems with long-range order, which, however, correlate more weakly than perfectly ordered crystals [10]. Hyperuniformity is a relatively new concept in statistical physics based on the long-range order in the material [28]. For disordered many-particle systems, such as an ideal gas, the variance of density (or of the number of points representing molecules per unit volume or area) is proportional to the volume (in the 3D case) or to the area (in the 2D case) of the system. An equivalent definition implies that the so called “structure factor” which characterizes correlation vanishes at the long-wavelength (small wavenumber, corresponding to large distance) limit, $lim\;s\left(k\right)=0$ or $s\left(k\right)\sim {\left|k\right|}^{\gamma }$ for k→0, where *k* is the wavenumber and γ is an exponent. That means that there is no correlation between the occurrence of the points separated by a long distance, $\frac{1}{k}$. For 0<γ<1, the long-range correlation vanishes faster than for a crystalline pattern, but slower than for a completely random set of points (i.e., γ=1) [10,28]. For a set of *N* points at positions ${\vec{r}}_{j}$, the microscopic number density is $\rho \left(\vec{r}\right)=\sum_{j=1}^{N}\delta \left(\vec{r}-{\vec{r}}_{j}\right)$. The Fourier transform is $\hat{\rho }\left(\vec{k}\right)=\sum_{j=1}^{N}e^{-i\vec{k}\cdot {\vec{r}}_{j}}$. The structure factor $S\left(\vec{k}\right)=\frac{1}{N}\left|{\hat{\rho }\left(\vec{k}\right)}^{2}\right|$ characterizes density fluctuations at the wavelength $\lambda =\frac{2\pi }{k}$.

## 2. Materials and Methods

### 2.1. Voronoi/Shannon Entropy

The research was conducted with a MATLAB R2022a (MathWorks, Inc., Natick, MA, USA) program implementing the following algorithm. First, 1000 random points were generated (uniformly distributed in the square [0, 1]^2^). This random pattern was used for the study. Next, the Voronoi tessellation/diagram was constructed. After that, a random area with a radius *R* was selected. This step was repeated many times, and the position of the center and the radius were varied, with 25 calculations at different center points for each radius. The Voronoi tessellation for points inside the circle is created considering only the polygons which were completely inside the circular area. The value of the VE was calculated in accordance with Equation (1). A statistical analysis of the results was performed.

**Figure 1:**
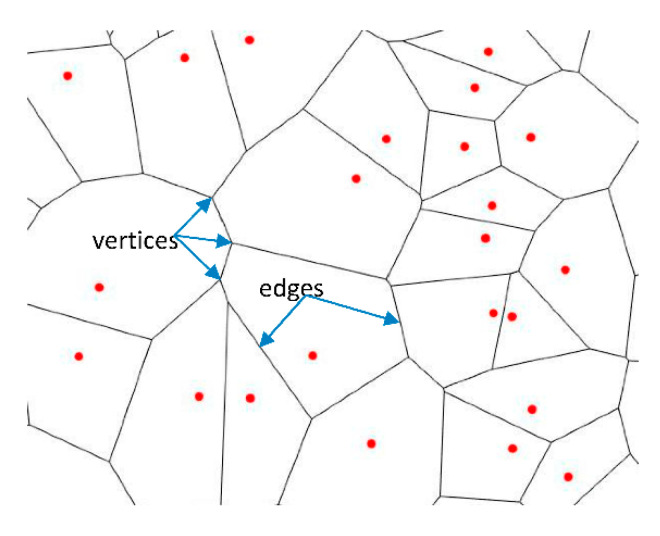
Example of the Voronoi tessellation on a set of points. Red points represent seeds or nuclei [2]. Blue arrows point to the edges of polygons.

The first step of calculating the VE of a set of points on a 2D surface is the generation of the Voronoi Tessellation. A Voronoi tessellation is built of polygons with the number of edges from *n* = 3 to *n* → ∞, although usually, the number of edges is small; thus, the maximum observed number of edges in a random system that has been reported in the literature is *n* = 15 [14,29]. The relative fraction of polygons with *n* edges is given by $0\leq p_{n}<1$. As it was discussed above, from the Euler topological equation, assuming that three edges meet at every vertex while each edge links two vertices (Figure 1), we find that the average number of edges is $\langle n\rangle =6$, given that each edge belongs to two polygons [2].

The Voronoi entropy was calculated with the moduli of the software developed at the Department of Physics and Astronomy at the University of California, Irvine (https://web.archive.org/web/20220611062558/https://www.physics.uci.edu/~foams/do_all.html accessed on 8 December 2023).

### 2.2. Long-Range Correlations

We followed the approach from Refs. [10,28,30,31], which present results for hyperuniform systems with partially constrained collective variables [10] and systems of particles interacting with stealthy pair potentials [30,31]. In such systems, the parameter γ characterizing the long-wavelength limit behavior of the autocorrelation function (ACF), C(R), can be introduced as the ratio of the number of constrained degrees of freedom to the total number of degrees of freedom [10]. Note that the hyperuniformity exponent is usually defined as the scaling exponent of the number variance of the system as a function of window size, based on which all hyperuniform systems can be classified into three categories [30,31]. The stealthy systems analyzed here possess the value of exponent equal to two irrespective of the value of the parameter γ.

The ACF for a random process characterizes the correlation of a parameter, such as the density $\rho \left(x\right)$ at a spatial distance *τ*, where *x* is the horizontal coordinate. The ACF is defined by correlating two points $\rho \left(x\right)$ and $\rho \left(x+\tau \right)$ separated by the distance τ

(3) $$C\left(\tau \right)=\frac{1}{LR_{q}^{2}}\int_{0}^{L}\left[\rho \left(x+\tau \right)-\bar{m}\right]\left[\rho \left(x\right)-\bar{m}\right]dx\;$$

where *L* is the length of the profile, $\bar{m}=\frac{1}{L}\int_{0}^{L}\rho \left(x\right)dx$ is the mean value of $\rho \left(x\right)$, and $R_{q}^{2}$ is the standard deviation needed for normalization purposes [32]. Equation (3) can easily be generalized for the 2D or 3D case.

The decay of the ACF in the long-range (large wavelength) limit may be exponential, $C\left(\tau \right)\sim e^{-\frac{\tau }{\beta }}$, where β is the correlation length or the decay may follow the power law,

(4) $$C\left(\tau \right)\sim \tau^{1-\gamma }$$

For a perfect pattern, γ=1, and the correlation remains finite in the long-range limit. On the other hand, for a completely random process, γ=0.

For a point process, the density distribution can be defined as $\rho \left(\vec{r}\right)=\sum_{j=1}^{N}\delta \left(\vec{r}-{\vec{r}}_{j}\right)$, where $\delta \left(\vec{r}\right)$ is the Dirac delta-function. The exponent γ=1−α is determined from the long wavelength limit (|k|→0) behavior of the structure factor $S\left(\vec{k}\right)\sim {\left|k\right|}^{\alpha }$. While γ=0 for a random pattern, and γ=1 for a perfect pattern (crystalline material), for a hyperuniform system, 0<γ<1, thus γ characterizes the suppression of large-scale density fluctuations.

## 3. Results

### 3.1. The Convergence of VE for a Poisson 2D Random Set Process

**Figure 2:**
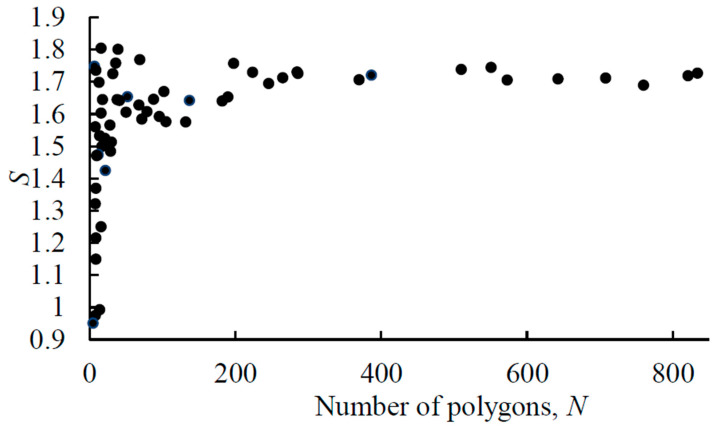
Voronoi entropy vs. the number of polygons in the Voronoi diagram (*n*) for a random set of seeds. The values shown for 60 random patterns generated with MATLAB having from 5 to 834 points.

The uniformly distributed random set of points in a square is often referred to as the Poisson point process. As it was discussed in the preceding section, the value of *S* = 1.690 ± 0.001 is considered as the average value of the entropy of a Voronoi random set of points. At the same time, the value *S* = 1.690 ± 0.001 is not the maximum possible value of the VE. Both random and ordered patterns can have even higher VE values. Figure 2 presents the calculated VE for a random point process as a function of the number of seed points. It is seen that with a small number of points *n* in a random pattern, the Voronoi entropy can both exceed and be significantly lower than the value characteristic of a random pattern.

For now, let us ignore the topological constraints typical for the Voronoi tessellations under the standard assumptions, such as the average number of edges $\langle n\rangle =6$ for tessellations with three edges per vortex. The maximum possible Voronoi entropy for a pattern of *N* types of polygons is achieved when there is an equal number of all types of polygons in the Voronoi tessellation. If there are *a* polygons of each type, then the total number of polygons is *aN*, so the maximum value of the Voronoi entropy is

(5) $$S_{vor}=-\sum P_{i}\ln P_{i}=-\sum \frac{a}{aN}\ln\frac{a}{aN}=-\sum \frac{1}{N}\ln\frac{1}{N}=\ln N$$

Note, however, that Equation (5) is true for a very special tessellation comprising *N* triangles, *N* quadrangles, … *N* polygons with *N* sides, and it states that VE grows unrestrictedly logarithmically with *N*, i.e., $S_{vor}\approx lnN$. For tessellations with $\langle n\rangle =6$, the VE can be estimated assuming there is equal number of polygons from *n* = 3 to *n* = 9 (with average $\langle n\rangle =6$) as $S_{vor}^{max}=-\sum_{i=3}^{9}P_{i}\ln P_{i}=-\ln\frac{1}{7}≅1.946$.

**Figure 3:**
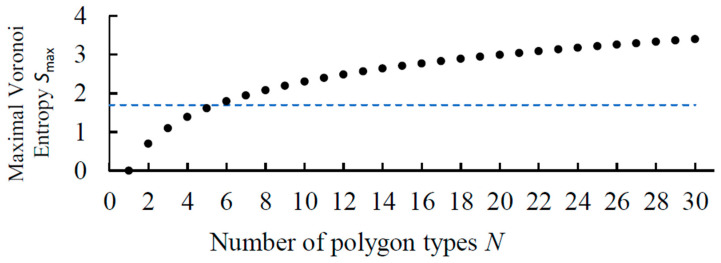
Maximum Voronoi entropy vs. the number of polygon types in a pattern, *N*. A dashed line shows the value for a random pattern.

The dependency of the maximum Voronoi entropy on the number of polygon types in the Voronoi tessellation is shown in Figure 3. The plotted function grows proportionally to the logarithm of the number of polygon types following Equation (5). For each fixed number of polygon types, there is a maximum Voronoi entropy. For example, if we have a Voronoi diagram consisting of only 6 polygons, and all these polygons are of different types (one triangle, one quadrangle, one pentagon, etc.), then the Voronoi entropy of this diagram is *S* = 1.79. As we can see, this value exceeds the value typical for a random pattern. In addition, Figure 3 suggests that the value *S* = 1.69 can only be obtained in patterns that are built from six or more different types of polygons.

From the computational modeling presented in Figure 2, it is observed that with an increase in the number of polygons in the pattern, the fluctuation of VE values decreases. Thus, for the number of polygons of about *n* ≈ 10, entropy can take values in the range between *S* = 1 and *S* = 1.8, while for the number of polygons of about *n* ≈ 100, entropy fluctuates within the range between *S* = 1.57 and *S* = 1.75.

Given that at least six types of polygons are needed to attain the asymptotic value of *S* = 1.69, the next question to consider is at what number of seed points on a 2D plane, the Voronoi diagram will include at least six types of polygons? In other words, which sample size of random points is needed to obtain the value of the VE typical for a random pattern?

**Figure 4:**
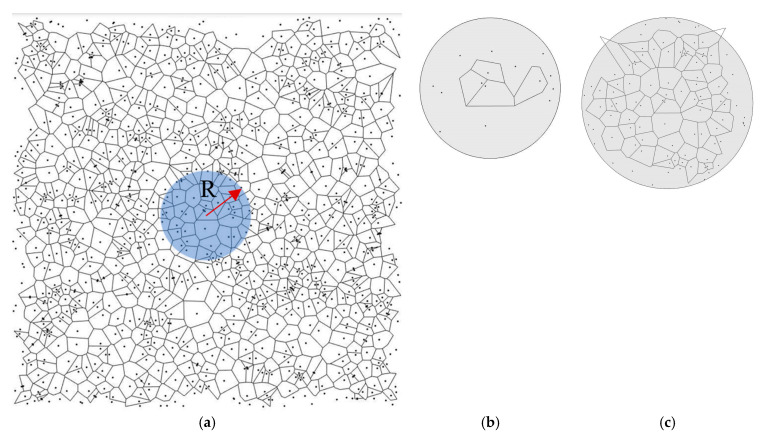
(**a**) Schematic of the method of determining the critical size of *R*. The Voronoi tessellation based on seeds (black dots) studied within the circular area *R*. (**b**) *Limit 1*: *R* ≈ 2.5; *VE* = 0 ÷ 1.6; (**c**) *Limit 2*: *R* ≈ 5.5; *VE* = 1.667.

To find the required sample size experimentally using computer simulation, let us consider a Voronoi diagram constructed on 1000 points, the coordinates of which are generated by a random number generator in the MATLAB program (Figure 4a). Let us rephrase the question posed differently: what size of the region of radius *R* in the diagram (Figure 4a) is necessary to characterize the random pattern using the Voronoi entropy. The radius *R*, as a dimensionless quantity, will be measured by the average distances between points *L_average_*. Let us assume that the coordinates of the points are in the interval [0, 1], the area occupied by the points is equal to unity and the average distance between the points is *L*_average_ = $\frac{1}{\sqrt{m}}$, where *m* is the number of points per unit area, in our case it is equal to 1000. The radius of the region under consideration cannot exceed half the interval [0, 1]. For example, it can be equal to *r* = 0.25. Then, expressed in average distances between the points, it will be equal to *R = r*/*L*_average_ = *r* $\sqrt{m}$ = 0.25$\sqrt{1000}$ ≈ 8.

**Figure 5:**
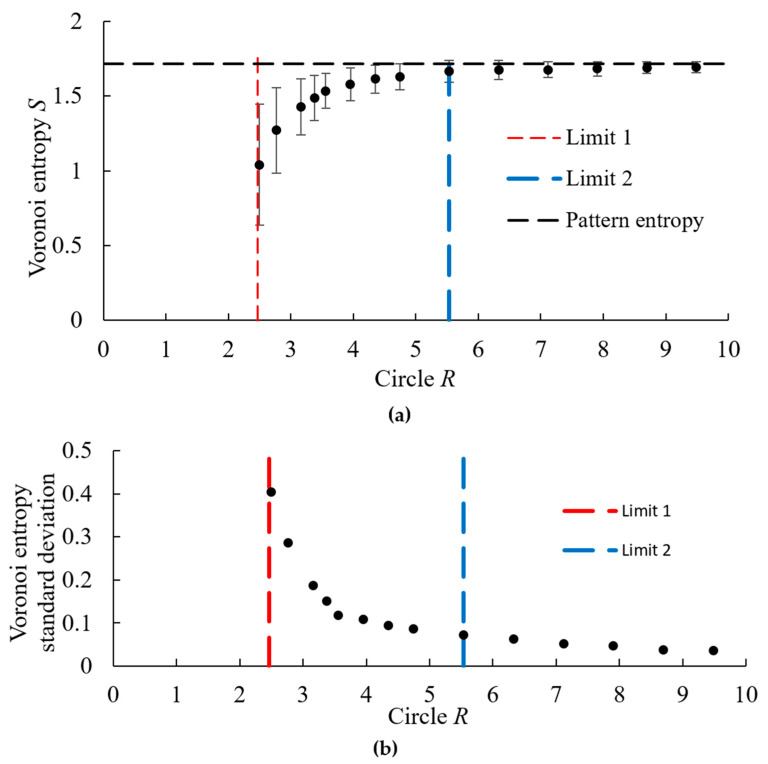
(**a**) The dependency of VE and (**b**) of the VE variance (standard deviation) on the radius, *R*. The radius is normalized by the average distance between seed points.

A random pattern of *n* = 1000 points was created and the Voronoi diagram was built. The value of the VE for the entire pattern was calculated using Equation (1) and found as *S* = 1.7158, which is greater than the theoretical value of the random point process, *S* = 1.690. After that, we calculated VE for circular fragments of the diagram having different radius *R* as well as the standard deviation of the VE (Figure 5a,b). For each value of the radius, 20–30 experiments were carried out having different centers but using the same random distribution of points. Only polygons that were completely inside the circle were considered for that purpose.

Patterns in areas with small radii contained the number of points smaller than a certain minimum critical number. For such patterns, it was not possible to build a Voronoi diagram due to an insufficient number of complete polygons inside the circle. As a result, we identified two critical values of the size of the Voronoi diagram. We called them *Limit-1* and *Limit-2* (Figure 4b,c).

The *Limit-1* is close to *R* ≈ 2.5, and it is the minimum radius of the region under consideration, for which it is possible to construct a Voronoi diagram. This radius corresponds to *n* = 16 ± 6 points in a circle. For the radii smaller than *Limit-1*, it was not always possible to create the Voronoi diagram. In other words, *Limit-1* is the lower limit at which VE is defined. For such small patterns corresponding to *Limit-1*, the average VE value was *S* = 1.04 ± 0.4 and it fluctuated within the range from *S* = 0 to *S* = 1.6.

With an increasing radius of the area, the VE grew. The value of the second limit, the *Limit-2*, corresponds to the saturation of the VE, and it was close to *R* ≈ 5.5. At this radius, the Voronoi entropy reaches the level characteristic of the random pattern. We can say that this is the minimum radius of the region that characterizes this pattern. For the considered pattern, *Limit-2* corresponds approximately to *N_r_* ~ 96 ± 6 points in a circle with radius *R*. In this case, the deviation from the Voronoi entropy of the entire pattern is less than 5% (1.716 − 1.667 = 0.049).

The obtained values of *N_r_* and *R* can be used for the study of the deviation of the patterns from the random homogeneous Poisson point process using the following procedure:

First, on the set of *N_r_* points (or on the region of radius *R*), a Voronoi tessellation is conducted and the value of VE is calculated, which is then compared with that of a random pattern.

**Figure 6:**
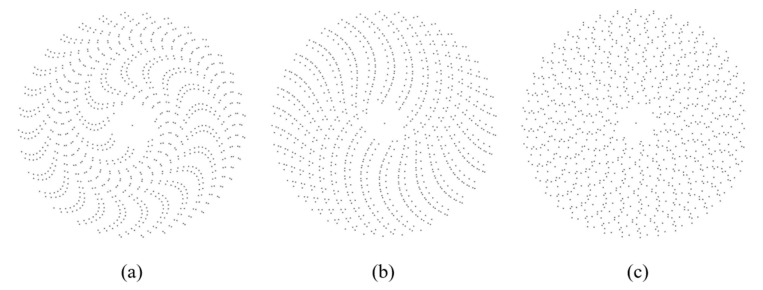
Patterns of points on Archimedes spiral with (**a**) S = 1.01, (**b**) S = 1.69, (**c**) S = 1.82.

It is noted that a deviation of the value of VE from *S* = 1.690 ± 0.001 for a higher or lower value does not necessarily mean higher or lower orderliness. Figure 6 shows three ordered point patterns obtained from the Archimedes spiral [33]. We can see that ordered patterns can have values of VE both higher and lower than 1.69 (Figure 6a,c). Moreover, an ordered pattern can have the VE value of *S* = 1.69. This suggests that in this case, VE is a measure of the diversity of the pattern (the Shannon diversity index), which characterizes the deviation from the hexagonal pattern.

### 3.2. VE of Hyperuniform 2D Datasets with Long-Term Order

**Figure 7:**
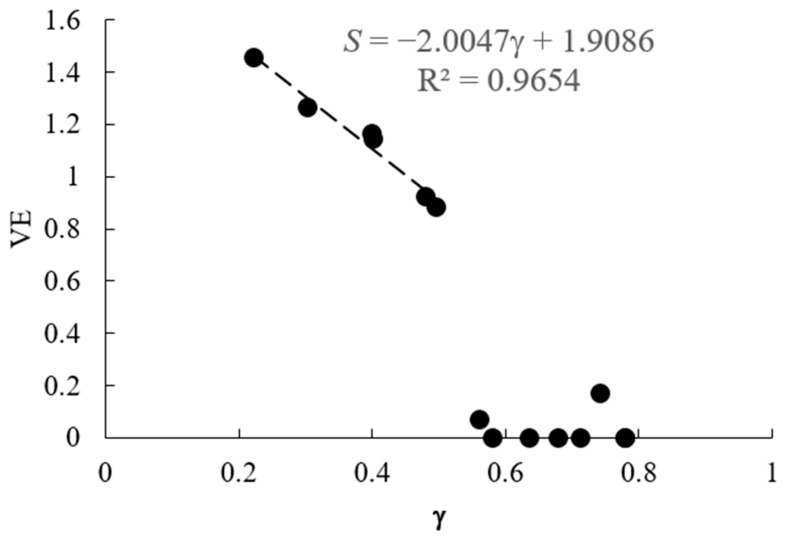
Correlation between the parameter *γ* and VE.

VE was calculated for 14 hyperuniform sets of points obtained from Uche et al. (2004) [10] and from Zhang et al. (2015) [30,31]. The correlation of the VE and the dimensionless parameter *γ* is presented in Figure 7.

It is observed that there is a strong negative correlation between the VE and the exponent parameter up to the value of γ≈0.5. With further increase, saturation happens, and VE abruptly drops to values close to *S* = 0. The coefficient of determination for the six data points before the saturation is *R*^2^ = 0.9654. Note that six data points is statistically weak, so the data should be interpreted with caution.

**Figure 8:**
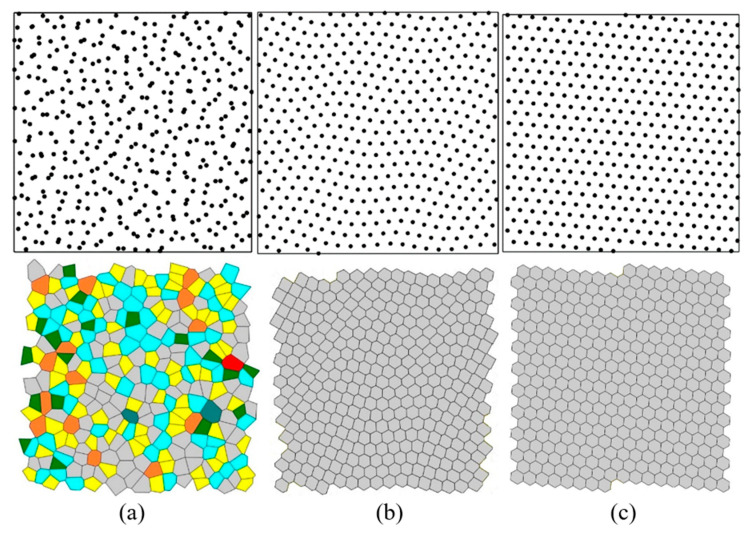
(**a**) Random *γ* = 0.22, *S* = 1.46, (**b**) Wavy Crystalline *γ* = 0.58, *S* = 0, and (**c**) Crystalline *γ* = 0.78, *S* = 0.

To explain this behavior, one can consider three examples corresponding to *γ* = 0.22 (*S* = 1.46), *γ* = 0.58 (*S* = 0), and *γ* = 0.78 (*S* = 0) [10]. Figure 8 shows the arrangement of points and the Voronoi tessellation for these three cases. The computer program marks polygons with different numbers of vertices by different colors: rectangles (green), pentagons (yellow), hexagons (gray), heptagons (blue), octagons (brown), enneagons (cyan), and decagons (red).

The first case corresponds to a random point distribution, so the value of *γ* = 0.22 is low, while the value of *S* = 1.46 is high. The last case corresponds to a periodic crystalline pattern, so the value of *γ* = 0.78 is high, while the value of S = 0 is at minimum. The second case corresponds to the “wavy crystalline” pattern, so that the value of γ = 0.58 is lower than that for the perfect crystalline, γ = 0.78; however, the Voronoi tessellation results in all polygons being hexagons (although of slightly different sizes). Hence the VE is again at minimum, *S* = 0, which explains the observed saturation.

The abrupt change in VE at *γ* ≅ 0.5 resembles the behavior of the true thermodynamic entropy under the so-called entropy-driven phase transitions when the phase transition is solely driven by the increase in entropy, as it takes place in colloidal crystals [34]. Generally, the VE is very different from the Boltzmann entropy [4]; however, in our research, it demonstrated the properties of thermodynamic entropy.

**Figure 9:**
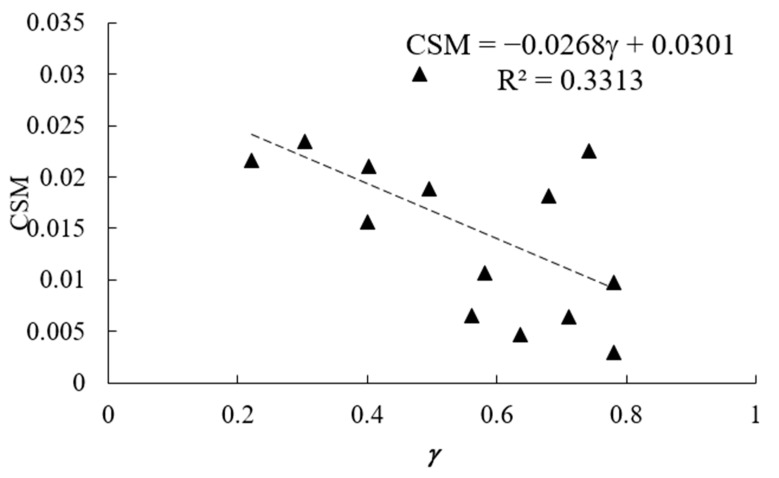
Correlation between the hyperuniformity exponent *γ* and CSM.

For comparison, the values of the CSM were also calculated using a Matlab program, as *ψ* = 0.0216 for the random (Figure 8a), *ψ* = 0.0107 for the wavy crystalline (Figure 8b), ψ = 0.0030 for the crystalline case (Figure 8c); thus, confirming that the crystalline case is more symmetric. Note that the wavy crystalline pattern, depicted in Figure 8b, follows the predictions of the Euler equation being constructed from hexagons only. The correlation between the CSM and *γ* is presented in Figure 9. The value of the coefficient of determination is *R*^2^ = 0.3313.

## 4. Discussion

Voronoi tessellation is based on the short-range orderliness of the system of points since the number of edges in a polygon depends only on the neighboring polygons. One could expect that it does not capture a long-range order.

The VE is a kind of Shannon entropy applied to the distribution of polygons emerging from the Voronoi tessellation. However, VE possesses specific properties, arising from the geometry of the entire Voronoi tessellation. Any Voronoi tessellation of any planar figure results in a certain distribution of the polygons’ probabilities, which is dictated by the topological constraints of forming the planar figure by the polygons. This situation is different from many typical cases of application of the Shannon entropy. For comparison, the Shannon entropy of a text consisting of *m* alphabetic letters (*m* = 26 for English) is given by a formula similar to Equation (1)

(6) $$H=-\sum_{n=1}^{m}p_{n}\ln\left(p_{n}\right)$$

where $p_{n}$ is the relative frequency of the *n*-th letter in the text. In the ideal case of a random text with the equal frequency distribution of letters, $p_{n}=\frac{1}{m}$, the value of Shannon entropy is at maximum, $H_{max}=\ln\left(m\right)$.

However, the situation is different with the VE, since the polygons are not completely independent from each other, since the tessellation has to keep the general topological properties of the entire tessellation untouched (those, prescribed by the Euler equation). Being a tessellation in one plane constrains the fractions of polygons in a non-obvious way. Therefore, besides pure local short-range interactions, long-range constraints are reflected in the resulting value of the VE. This is the reason why VE correlates with the long-range parameter, such as the critical exponent *γ*.

On the other hand, the VE does not depend on the size or area of polygons. Instead, it takes into account only the number of edges. This explains the saturation (or the abrupt drop) of the correlation dependency in Figure 2 corresponding to the “wavy crystalline” arrangement of points.

Note also that the wavy crystalline phase cannot be distinguished from the perfect crystalline phase with VE. One might generalize VE to distinguish these two phases, for example, including a joint distribution of the number of edges and the length of perimeters, or some measure of the anisotropy of Voronoi cells. Potentially, VE can be applied to the study of other types of correlated disordered point configurations, such as hard-particle fields and systems with Random sequential adsorption [35].

## 5. Conclusions

We studied the asymptotic maximum value of the VE for a 2D random point process, which is equal to *S* = 1.690 ± 0.001, and the rate of convergence of the VE to the asymptotic value with increasing number of polygons in the Voronoi tessellation. We found that for the number of polygons of about *n* > 100, the VE range reaches its saturation values, namely, between *S* = 0 for an ordered set of seed points and *S* = 1.69 for a random set of seed points. For circular regions with the dimensionless radius *R* measured in the number of average distances between points we identified two limiting radii. *Limit-1* with *R* ≈ 2.5 and *n* = 16 ± 6 points is the minimum radius, for which it is possible to construct a Voronoi diagram. *Limit-2* with *R* ≈ 5.5 and 96 ± 6 points is the radius at which the VE reaches the saturation level. We also discussed examples of ordered and random seed point patterns, for which the values of VE exceed the asymptotic value of *S* > 1.69.

We also calculated the VE and the CMS for hyperuniform sets of points with the imposed long-range geometrical correlation and compared the VE and CMS to the values of exponents γ of collective density variables characterizing long-range correlations in the system. We found that the VE correlates with the exponents γ up to a certain saturation level (γ ≅ 0.6), after which the value of the VE abruptly falls to *S* = 0. We explain this phenomenon by the fact that the distribution of polygons by the number of edges in a given pattern is constrained by being a tessellation within a plane (namely, a surface possessing given topological properties). Therefore, besides pure local short-range correlations, long-range topological constraints are reflected in the resulting value of the VE. This becomes clear if we consider that just the seminal Euler topological Formula predicts that the most abundant polygon in the random Voronoi tessellation is a hexagon. The saturation is explained by the lack of sensitivity of the VE to the size/area of the polygons. This hypothesis will be checked in our future investigations, devoted to the study of the patterns built on a diversity of surfaces.

## Figures and Tables

**Table 1:** Voronoi Entropy values obtained based on data published in [11].

Authors	Voronoi Entropy
Crain, 1978 [12]	1.689266
Hinde & Miles, 1980 [13]	1.690754
Kumar & Kurtz, 1993 [14]	1.689021
Tanemura, 2003 [15]	1.690262
Brakke, 2005 [16]	1.69031

## Data Availability

The data presented in this study are available on request from the corresponding author.

## Abbreviations

The following abbreviations are used in this manuscript:

ACF	Autocorrelation Function
CSM	Continuous Symmetry Measure
VE	Voronoi Entropy
